# Radiobiology and Reproduction—What Can We Learn from Mammalian Females?

**DOI:** 10.3390/genes3030521

**Published:** 2012-08-27

**Authors:** Aurora Ruiz-Herrera, Francisca Garcia, Montserrat Garcia-Caldés

**Affiliations:** 1 Departament de Biologia Cellular, Fisiologia i Immunologia, Universitat Autònoma de Barcelona, Campus UAB, 08193, Cerdanyola del Vallès, Barcelona, Spain; 2 Institut de Biotecnologia i Biomedicina (IBB), Campus UAB, 08193, Cerdanyola del Vallès, Barcelona, Spain; 3 Unitat de Cultius Cellulars, Servei de Cultius Cellulars, Producció d'Anticossos i Citometria (SCAC), Universitat Autònoma de Barcelona, Campus UAB, 08193, Cerdanyola del Vallès, Barcelona, Spain; E-Mail: francisca.garcia@uab.cat

**Keywords:** meiosis, oocytes, fertility, ionizing irradiation effects, radiation-sensitivity, DSBs, genome instability

## Abstract

Ionizing radiation damages DNA and induces mutations as well as chromosomal reorganizations. Although radiotherapy increases survival among cancer patients, this treatment does not come without secondary effects, among which the most problematic is gonadal dysfunction, especially in women. Even more, if radio-induced DNA damage occurs in germ cells during spermatogenesis and/or oogenesis, they can produce chromosomal reorganizations associated with meiosis malfunction, abortions, as well as hereditary effects. However, most of our current knowledge of ionizing radiation genotoxic effects is derived from *in vitro* studies performed in somatic cells and there are only some experimental data that shed light on how germ cells work when affected by DNA alterations produced by ionizing radiation. In addition, these few data are often related to mammalian males, making it difficult to extrapolate the results to females. Here, we review the current knowledge of radiobiology and reproduction, paying attention to mammalian females. In order to do that, we will navigate across the female meiotic/reproductive cycle/life taking into account the radiation-induced genotoxic effects analysis and animal models used, published in recent decades.

## 1. Introduction

Ionizing radiation (IR) damages DNA and induces genetic mutations as well as chromosomal reorganizations, that result in single-strand breaks (SSBs) and/or double-strand breaks (DSBs). DSBs constitute a particular hazard to cells given that, if persistent, they can lead to carcinogenesis. In mammalian cells, different pathways in response to DNA damage maintain genome stability controlling either the cell cycle or the repair of the damage itself. These mechanisms are known as the DNA damage response (DDR), which includes several pathways that are selectively activated depending on the genetic insult (reviewed in [1]). Especially relevant to radiobiology are the mechanisms that detect and repair SSBs and DSBs. The DDR can be divided into a series of distinct pathways depending on the type of DNA lesion they process (review in [1]). SSBs are repaired basically by the base excision repair (BER) mechanism with the implication of several proteins such as PARP1, XRCC1 or LIGASE 3. DSBs, on the other hand, are repaired through two main mechanisms: (i) homologous recombination (HR), which requires large regions of homology, and (ii) non-homologous end joining (NHEJ) [2]. NHEJ is active preferentially during the G_1_ to early S phase of the cell cycle whereas HR takes place in late S and G_2_ phases. Both mechanisms are implicated in the reparation of radio-induced DSBs given that mammalian cells defective in proteins involved in these processes (*i.e.*, DNA-PKcs or RAD54) have been shown to be hypersensitive to ionizing radiations [3,4,5].

Most of our current knowledge of ionizing-radiation genotoxic effects comes from *in vitro* studies performed in somatic cells. But if radio-induced genetic insults occur in germ cells, during spermatogenesis and/or oogenesis, chromosomal reorganizations can appear, and it is well known that they are associated with meiosis malfunction and abortions as well as hereditary effects. Therefore, our need for radiobiology studies in the germ line is beyond question. Especially relevant for reproductive studies are the proteins implicated in the recognition and repair of DSBs by homologous recombination (HR) that occurs during the meiotic prophase I. At this stage, HR is essential for the proper disjunction of homologous chromosomes, which is triggered by programmed DSBs, the formation of which is catalyzed by the DNA topoisomerase-II-related protein Spo11 [6]. In addition to Spo11, DSB formation requires other proteins [7]. The broken ends are processed by 5' to 3' degradation, and the resulting 3' single-stranded ends invade the homologous chromosome, which results in the formation of a heteroduplex. The resulting single stranded DNA (ssDNA) forms the substrate for subsequent strand invasion, which is catalyzed by the two recA homologs Rad51 and Dmc1. Some proteins involved in the recombination process have been identified in the last few years. The replication protein A (RPA) is a component of the transitional meiotic nodules [8] while MLH1 is a marker of crossovers (COs) events [9]. The meiotic prophase I is under the regulation of checkpoint systems that recognize those chromosomes that remain unpaired at the pachytene stage and lead to its silencing through the recruitment of proteins such as BRCA1 and ATR [10]. Therefore, germ cells, depending on the cell cycle, would contain detectable levels of different proteins involved in the recognition and repair of DSBs by HR (ATR, ATM, RAD51, BRCA1, BRCA2, MSH2 and MSH3, among others). The presence of these proteins might make meiotic cells less sensitive to the DSBs induced by IR than other cell types. However, at this stage, it is not known how these proteins interplay when radio-induced genetic insults occur during the meiotic prophase I.

Radiotherapy has tremendously increased survival among cancer patients. However, this treatment does not come without secondary effects, among which the most problematic one refers to gonadal dysfunction, especially in women [11,12]. Furthermore, although now it is possible to estimate, based on mathematical models, the dose of fractionated radiotherapy (Gy) at which premature ovarian failure occurs immediately after treatment with IR [12], our knowledge of the genotoxic effects is still very poor. Therefore, wider knowledge of the genotoxic and gonadal effects of ionizing radiation upon the female function and the possibility of treatment or prevention of the ovarian lesion would mitigate some of the consequences of these therapies in cancer patients. There are recent reports that suggest a link between resistance to radiotherapy and altered DDR activity in cancer stem-cells and tumor-initiating cells [13], so new targets for therapy can be developed in the near future. With this big picture in mind, here we review the current knowledge of radiobiology and reproduction, paying special attention to mammalian females. We will navigate across the female meiotic cycle, focusing on the radiation-induced genotoxic effects analysis and animal models used in the last three decades, analyzing what we can learn from mammalian females.

## 2. Female Gametogenesis: Its Complexity and Uniqueness

**Figure 1:**
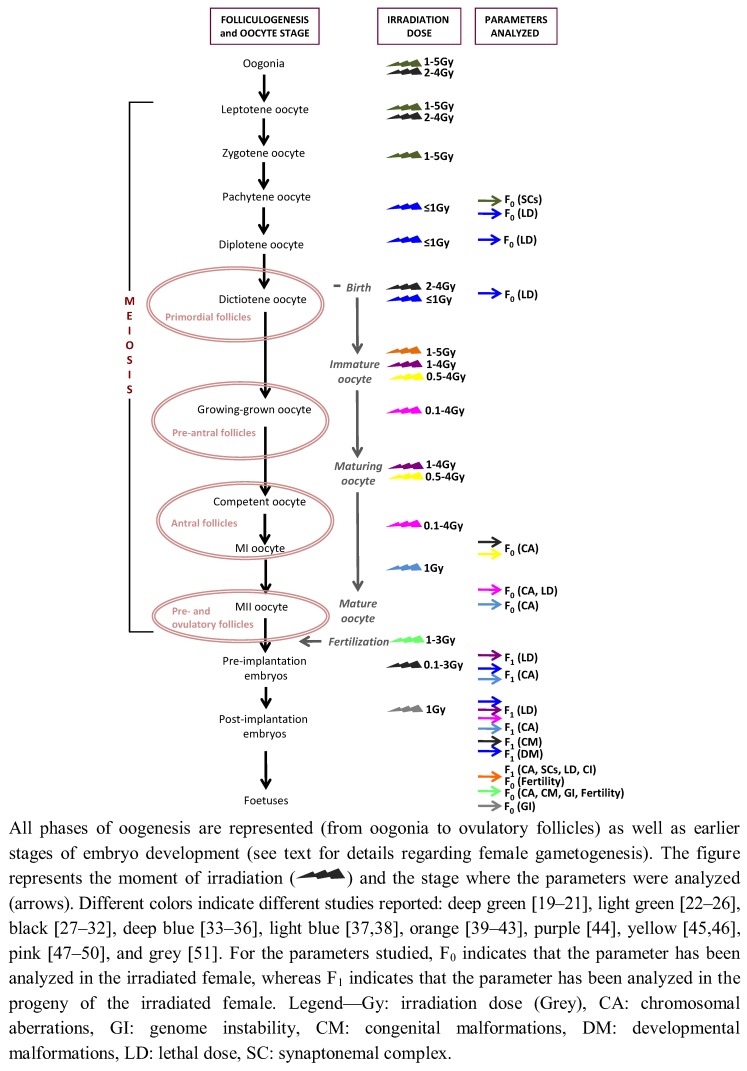
Schematic representation of the radiation experiments *in vivo* performed in mammalian females considering the folliculogenesis/oocyte stage, the irradiation dose and the parameters analyzed.

Gametes are produced during gametogenesis through a meiotic program, which involves two successive rounds of cell division (MI and MII) that follow a single round of DNA replication. In mammals, oogenesis is a complex physiological process that differs from spermatogenesis in several ways, such as gametes morphology and differentiation, place and timing, among others. While spermatogenesis produces a relatively small motile gamete, the oocyte is a bigger cell that contains all the materials needed to initiate and maintain metabolism and development. Whereas spermatogenesis occurs during the whole active life of an adult, thus males have the ability to produce spermatocytes continuously, oogenesis initiates early in development, becomes arrested at the moment of birth and resumes at the puberty stage (Figure 1). This intrinsic complexity has, as a result, meant that female mutation data are scarce in the literature. From the few data available the general view that can be extracted is the existence of gender differences in the induction of chromosomal aberrations and gene mutations [14,15].

In humans, around the 22nd embryonic day, primordial germ cells migrate to the embryonic gonads. Since then and until the 6th month of pregnancy, germ cells enter meiosis, complete meiotic prophase I, and arrest at the dictiotene stage until puberty begins. At the moment of birth, the ovaries of a human female contain approximately 2 × 10^6^ immature, primordial follicles [16], each of which contains an immature primary oocyte. In mice, two-thirds of the oocytes enter apoptosis after birth, while the remaining oocytes separate from the cysts into single cells that become surrounded by granulose cells to form primordial follicles [17]. In fact, it has been reported that, in the mouse C57BL/6 strain, the mean number of primordial follicle numbers and oocytes (±s.e.m.) in Day 1 after birth (a.b.) ovaries was 7,924 ± 1,564 and declined significantly to 1,987 ± 203 in Day 7 a.b. ovaries due to programmed cell death [18]. Then, primordial follicles are gradually recruited to primary and secondary pre-antral follicles, a process known as folliculogenesis. During this stage, both oocytes and granulose cells grow and mature in a synchronic way, establishing cell communications and, therefore, they can be considered as a functional syncytium. After puberty, with the first menstruation, a number of follicles enter into a growth pattern that will end in cell death or in ovulation (the process where the oocyte leaves the follicle). In humans, folliculogenesis lasts for approximately 375 days, being a process that operates regularly. This means that at any time of the female reproductive life the ovary contains follicles in all stages of development, and ends when a mature oocyte departs from the ovulatory follicle during ovulation. Roughly, these stages include, following a sequence in time: primordial follicles, pre-antral follicles, antral follicles (also known as pre-ovulatory follicles) and ovulatory follicles (Figure 1). While the follicles are growing, the oocytes I grow and mature in parallel, which is a complex phenomenon that includes both nuclear and cytoplasm maturation. While the nuclear maturation implies that the oocyte progresses from diplonema to metaphase II (MII) stage, there are essential transformations at the cytoplasmatic level that prepare the cell to support fertilization and early embryonic development. Only when the oocyte is competent, is it released and ready for fertilization. Immediately after fertilization, the zygote (the fertilized egg) enters into a series of rapid mitotic divisions (cleavage) that ends with the formation of the blastocyst, which hatches from the *zona pellucida* and is ready for implantation. This pre-implantational period takes between 5–6 days in mice, and a little bit longer in humans. After implantation, organogenesis (the production of tissues and organs)/fetogenesis begins a process that ends at the moment of birth.

In any mammalian female at the moment of birth, immature oocytes (non-growing oocytes I, growing oocytes I and grown oocytes) are arrested at the dictiotene prophase I stage and can form primordial follicles, primary and/or multilaminar follicles or pre-antral follicles. During maturation, the oocyte is still arrested at diakinesi but *granulosa* cells grow and interact with the growing oocytes, gradually forming pre-antral follicles (Figure 1). The literature is sometimes misleading with the use of these terms, but we can consider that antral follicles (also known as pre-ovulatory follicles) contain maturing and mature oocytes I, whereas ovulatory follicles have mature and competent oocytes at the MII stage. In this sense, it is important to bear in mind that oocytes are blocked in prophase I during long periods of time. In humans, for example, some oocytes will be maintained in meiotic prophase I for many years. The challenging goal in this context is to conserve an oocyte without any defect to proceed to cycle. Also, during this long period, oocytes have high probabilities to suffer genetic insults, which, if repaired incorrectly, can induce permanent aberrations that can be propagated to the progeny. Evidence indicates that oocyte radiation-sensitivity depends on its developmental stage and its relation with folliculogenesis. So, this nomenclature is important, given that most of the irradiation studies, conducted mainly in rodents, have been focused on immature and mature/pre-ovulatory oocytes (Figure 1).

Mutational studies on the effects of ionizing irradiation in somatic cells have revealed that genotoxicity depends not only on the irradiation dose, but also on the cell type and cycle. Such studies have contributed enormously to our knowledge on how IR induces mutations and how they are repaired [1]. But if our goal is to study how radio-induced genetic insults affect reproduction and/or how they are propagated to the offspring, we need direct observations of the germ line. At this point, and despite its importance, the study of the genotoxic effect of IR on the germ line faces numerous methodological challenges, such as the intrinsic nature of oogenesis, animal manipulation, time-consuming protocols and the lack of an *in vitro ad hoc* methodology. The intrinsic nature of female gametogenesis, long periods (as in years) make it possible that errors induced by genotoxic agents occur during meiosis compromise fertility, the viability of the embryos and, eventually, induce genetic abnormalities that can be propagated in the offspring (so-called trans-generational effects). As stated earlier, meiotic prophase I occurs during the prenatal process of oocyte formation. This first stage of meiosis begins in the embryo and stops at birth, reactivating the signal to resume meiosis at the puberty. This complexity also makes the genotoxic effect of ionizing radiation difficult to evaluate in the female germ-line. Not all cells are equally sensitive to radiation damage; cells that divide rapidly and/or are relatively non-specialized, tend to show effects at lower doses of radiation than do those that have a longer cell cycle. It has been reported that, during oogenesis, the sensitivity of oocytes to genotoxic factors depends on the meiotic phase and the stage of folliculogenesis [52,53].

Another level of complexity is animal manipulation for *in vivo* experiments and the difficulty of extrapolating results among different species models. It has been suggested, for example, that the guinea pig is the best model for the study of radiation-induced genotoxic effects in the female germ-line [27] but as we will see herein, that would depend on the parameter analyzed (Table 1). Therefore, for any radiobiology study, it is of relevant importance to consider the cell type, age and/or developmental stage at the moment of the irradiation and the parameter considered, as well as the model species, in order to understand the reproductive implications of radiation damage.

**Table 1:** Summary of the results of genotoxic effects of ionizing irradiation on experimental models.

Species studied	Meiotic/Oocyte stage and irradiation dose	Parameters analyzed	Summary of results	Reference
mouse	Adult females	Locus-specific mutation at F_1_	(−)	[54]
	63 rads			
mouse	Ovaries	Cell killing	LD_50_: 0.15 Gy	[16]
	0.15 Gy	Fertility	Maximum 4 litters/female;	
			Early follicles RS > Larger follicles RS	
mouse	Mature and immature oocytes	Dominant lethality (pre and post-implantation mortality)	RS species and oocyte stage-dependent	[44]
	100-400 rads			
mouse	Pre-ovulatory oocytes	Chromosomal aberrations at MII	(+)	[37,38]
	22-600 rads		Mature oocyte RS > Immature oocyte RS	
	Adult females	Chromosomal aberrations at:		
	0.22, 0.66, 2 and 6 Gy	• MII	(+)	
		• 2-cell embryo	(+)	
		• 13.5-day embryo	(−)	
mouse	Mature and immature oocytes	Chromosomal aberrations at MI	Mature oocyte RS >Immature oocyte RS	[46]
	50-400 rads			
mouse	Immature oocytes	Chromosomal aberrations at dictionema	(−)	[55]
	400 rads			
mouse	Pre-ovulatory oocytes	Chromosomal non-disjunction at MI	(+) At higher dose	[56]
	0.05-0.80 Gy			
		Structural chromosome aberrations at MII	(+)	
mouse	Adult females	Dominant lethality	(+)	[57]
	108-504 rads	Developmental malformations	(+)	
			Immature oocyte RS > Mature oocyte RS	
mouse	Juvenile mice	Primordial oocyte killing	LD_50_: 6-7 rads	[58]
	6 and 7 rads			
mouse	Oocytes at dictionema	Chromosomal aberrations at one-cell embryos	(+)	[47]
	100-600 cGy			
mouse	Pre-ovulatory oocytes	Chromosomal aberrations at MI	(+)	[48]
	≤100 cGy			
mouse	Zygote stage	F_1_ chromosomal aberrations and micronuclei	(+/−)	[25]
	2Gy			
mouse	Immature oocytes	Chromosomal aberrations at MII	(+)	[50]
	0.1 and 0.2 Gy			
mouse	Fetal oocytes at 14, 16, and 17 days of gestation	SC anomalies at pachynema	Fragmentation stage-dependent	[21]
	2 Gy			
mouse	Pre-implantation stage	Developmental malformations	(+)	[31]
	≤3 Gy		LD_100_: 0.5 Gy	
	Oocytes within 1-4 weeks before ovulation	Developmental malformations	(+)	
	2 and 3 Gy			
mouse	Pre-ovulatory oocytes	Chromosomal aberrations at:		[49]
	1-4 Gy	• MII	(+)	
		• pre-implantation stages	(+)	
		• post-implantation stages	(−)	
mouse	Pre-implantation stage (2 h, 48 h, 72 and 96 h post-conception)	Developmental malformations and mortality	(+)	[32]
			RS irradiation stage-dependent	
	0.1-2.5 Gy			
mouse	Female zygote stage	Fertility alterations	(+)	[22]
	1 Gy	F1 developmental malformations	(+)	
mouse	Female zygote stage	Fertility alterations	(−)	[23,24]
	0.2 and 0.4 Gy	F_1_ developmental malformations	(−)	
		Trans-generational genomic instability (chromosomal aberrations)		
			(−)	
mouse	Pre-conception stage	Developmental malformations	(+) dose-dependent	[26]
	1, 2.8, and 3 Gy			
	Zygote stage	Developmental malformations	(+) dose-dependent	
	1, 2.8, and 3 Gy			
	Pre-implantation stage	Developmental malformations	(+) dose-dependent	
	1, 2.8, and 3 Gy			
	Zygote	Trans-generational genome instability (chromosomal aberrations)	(+)	
	500 mGy, 1,000 mGy and 2,000 mGy			
mouse	Post-implantation stage	Trans-generational genome instability (ESTR mutation frequencies)	(−)	[51]
	1 Gy			
mouse	Pre-conception stage	Trans-generational genome instability (polymorphism of DNA fragments)	(+) tissue-dependent	[59]
	0.5, 1 and 2 Gy			
mouse	Adult female	Transgenerational genome instability (ESTR mutation frequencies)	(−)	[60]
	1 Gy			
golden hamster	Mature and immature oocytes	Dominant lethality (pre and post-implantation mortality)	RS species and oocyte stage-dependent	[44]
	100-400 rads			
guinea pig	Mature and immature oocytes	Dominant lethality (pre and post-implantation mortality)	RS species and oocyte stage-dependent	[44]
	100-400 rads			
guinea pig	Mature and immature oocytes4 Gy	Dominant lethality (embryo mortality)	Mature oocyte RS > Immature oocyte RS	[45]
guinea pig	Oogonia and oocytes at leptonema	Fertility	(−)	[27,28,29,30]
	2 and 4 Gy			
	Oocytes at birth and at adulthood	Cell-killing	LD50: 4 Gy	
		Fertility	(−)	
	2 and 4 Gy			
	One-cell embryo stage	Developmental malformations	(+)	
	10, 50 and 100 cGy			
	Oocytes at birth	Chromosomal aberrations at MI	Mature oocyte RS > Immature oocyte RS	
	1 and 2 Gy		Nearly mature guinea pig oocyte RS > Nearly mature mouse oocyte RS	
guinea pig	Oocytes at different stages of folliculogenesis	Chromosomal aberrations	Immature but grown oocyte RS < Maturing oocyte RS < Mature oocyte RS	[23,24]
	4.0 Gy			
			RS of guinea pig oocyte ≠ RS of mouse oocyte	
Chinese hamster	Oocytes around ovulation	Chromosomal aberrations at diakinesi stage	(+)	[61,62,63]
	2 Gy		mouse oocyte RS = 2 × Chinese hamster oocyte RS	
Chinese hamster	Oocytes at pachynema	Cell-killing	RS meiotic stage-dependent	[33,34,35,36]
	1 Gy			
	Oocytes at diplonema-dictionema	Cell-killing	LD_100_: 1 Gy	
	1 Gy			
	Oocytes at pachynema and diplonema-dictionema	Developmental malformations	(−)	
		Chromosomal aberrations at 1-cell embryos		
	1 Gy		(−)	
rat	Ovary	Cell-killing	Primordial germ cell reduction (66%)	[64]
	1 Gy			
rat	Ovary	Cell-killing	LD_50_: 1 Gy	[16]
	1 Gy			
rat	Oogonia, oocytes at leptonema and zygonema	SC anomalies at pachynema	Fragmentation stage-dependent	[19,20]
		Cell killing		
	1, 2 and 5 Gy		(+) dose-dependent	
rat	Immature oocytes of pre-pubertal and post-pubertal females	Fertility alterations	Pre-pubertal oocyte RS < Post-pubertal oocyte RS (−)	[39,40]
		SC alterations of F_1_ female fetuses		
	1, 2 and 5 Gy			
rat	Primordial follicle oocytes	Fertility alterations	(−)	[41,42,43]
	5 Gy	F_1_ constitutional chromosomal aberrations	(−)	
		Trans-generational genome instability	(+)	
		Trans-generational sensitivity to chemical mutagen	Increased	

RS: radiation sensitivity; LD: lethal dose; SC: synaptonemal complex; Gy: Grey; F_1_: progeny of the irradiated female, MI: metaphase I, MII: metaphase II; (+): the parameter analyzed is affected by ionizing radiation; (−): the parameter analyzed is not affected by ionizing radiation.

## 3. Indicators of Radiation-Induced Genotoxic Effects

As stated earlier, radiation-sensitivity is a complex affair given that it depends on several factors, such as type of irradiation, total dose irradiated, time-interval over which dose is received, type of cell affected or the stage of cell division irradiated, among others. At the same time, and as far as we know, the mechanism by which radiation causes damage to cells is by ionization of atoms in the molecules, which can affect the genetic material (DNA) or associated proteins. Therefore, the direct or indirect indicators of this primary damage are variable, and in this sense the results can be uncertain and even contradictory. Overall, when IR affects the DNA, two different categories can be established; short term effects (such as effect on DNA: DSBs, SSBs, among others) and long terms effects (cell killing and aneuploidies, among others). Here, we will provide a general view of the parameters that have been used in the literature for the assessment of radiation-induced biological effects, reflecting the high complexity of the state of the art.

### 3.1. Cell Killing (Lethal Dose, LD)

In toxicology, the lethal dose (also referred to as LD_50_, lethal dose 50% or median lethal dose) is an indicator used as a measure of cell killing and fertility. The LD_50_ for a particular substance (*i.e.*, ionizing radiation) refers to the amount (mgr) necessary per weight (kg) to cause death in 50% of the population under study. Early studies used this measure of toxicological effects of ionizing irradiation in mouse [16,58], rat [16,19,20,64], Chinese hamster [33,34,35] and guinea pig [27,28,29,30]. Results have been heterogeneous depending on the species studied and the moment of the irradiation (oocytes and pachynema, diplonema-dictionema or at the moment of birth). Although studies are scarce, the observed trend is that LD is species-dependent.

### 3.2. Fertility Alterations

Fertility alterations such as oocytes depletion, ovarian failure and loss of reproductive potential have also been used as indicators of radiation-induced genotoxic effects only in a few numbers of reports (Table 1). This includes studies in mouse [16,22], guinea pig [27,28,29,30] and rat [39,40,41]. The results so far are contradictory but the general observed trend is that fertility alterations do not seem to be related to the species irradiated but rather to the irradiation timing. 

### 3.3. Developmental Malformations

Developmental malformations are anatomical or structural abnormalities observed at birth and may be caused by genetic factors or environmental alterations (or a combination of the two) that occur during prenatal development. Although genetic factors are the most common causes of developmental malformations, the underlying mechanisms are complex and not well known. Due to this complexity, only few authors have used developmental malformations as indicators of radio-induced insults (Table 1). At the same time, the results obtained so far (either positive or negative) can even be contradictory due to the great diversity of alterations that can be considered as “malformations”, as well as to different moments (pre- and post-implantation, for example) when observed.

### 3.4. Genetic Mutations

The DNA double-strand breaks (DSBs) produced by IR constitute a particular hazard to cells and can result in genetic mutations that can be evaluated. Several parameters are used to evaluate radiation-induced genetic mutations, and here we will review those that can directly affect the DNA (*i.e.*, dominant lethal effects, locus-specific mutations, chromosomal aberrations, genomic instability) or associated proteins (*i.e.*, synaptonemal complex). 

A parameter that has been traditionally used in toxicology has been the dominant lethal effects, (DL) [44,45,57]. DL events are genetic effects cause by any physical or chemical agent that causes embryonic or fetal death, indicating that the substance has affected germinal tissue. According to the OECD (Organization for Economic Co-operation and Development) guidelines for the testing of chemicals, “a dominant lethal mutation is one occurring in a germ cell which does not cause dysfunction of the gamete but which is lethal to the fertilized egg or developing embryo”. Therefore, DL causes embryonic or fetal death. They are generally accepted to be the result of chromosomal damage, but gene mutations and toxic effects cannot be excluded. Studies performed in golden hamster, mouse and guinea pig [44,45,57] have revealed that the radiation sensitivity obtained from evaluating DL is both species- and oocytes stage-dependent (Table 1).

Additionally, locus-specific mutations have been used to detect recessive mutations induced in diploid organisms: a strain that carries several known recessive mutations in a homozygous condition is crossed with a non-mutant treated strain in order to look for these recessive mutations in the progeny. This has been the case of mutations in genes controlling mouse fur color, a method initially developed by Russell and Russell [65] and applied later on. However, this method has been reported to have low mutation rates and therefore low sensitivity to detect trans-generational effects [66]. An alternative to overcome this limitation is the detection of *loci* containing repetitive sequence elements (expanded simple tandem repeat—ESTR—DNA *loci*), which are known to mutate at an extremely high frequency [67]. Jointly with chromosomal reorganizations, this has been the technique most widely used to detect trans-generational genotoxic effects of chemical and physical (*i.e.*, IR) agents, as we shall see later on.

When IR damages DNA and induces chromosomal anomalies through the formation of DSBs, such alterations can be stabilized through chromosomal rearrangements or capped by the addition of telomeric sequences, producing chromosomal aberrations. Revisions focused on different types of radiation-induced chromosomal reorganizations previously have been reported elsewhere [68], so here a brief summary is provided. As a general overview, chromosomal reorganizations induced by IR are mainly structural changes resulting in inversions (either paracentric or pericentric), translocations, fusions and fissions, among others, and to a lesser extent, numerical. These structural aberrations can be considered as (i) chromatid-type or (ii) chromosome-type. The former affects one of the sister-chromatids, whereas both sister-chromatids are involved in the latter. Both types of chromosomal aberrations have been used as reliable biomarkers for genetic radio-induced damage. When occurring in the germ line, chromosomal aberrations can lead to aneuploidy and/or constitutional aberrations in the offspring.

In a genome-wide broader scale, the increased rate of alterations produced by the effect of IR is known as radiation-induced genomic instability (RIGI) [69]. Genomic instability is a term initially used to describe a phenomenon that results in the accumulation of multiple changes required to convert a stable genome of a normal cell into an unstable genome characteristic of a tumor, and it is known that this can be caused by radiation [70]. RIGI can be measured by the formation of gene amplification, micronucleus formation and microsatellite instability, as well as chromosomal reorganizations, and its effects are dependent on the genetic background of the cell [71]. When inducing genetic abnormalities that can be propagated in the offspring, “trans-generational RIGI” can also be measurable as a biomarker for radio-induced genotoxic effects [41]. 

Radiation-induced genetic mutations can also be measured by analyzing the proteins associated with the DNA. In the case of the germ line, the synaptonemal complex (SC) has been used as a biomarker for genotoxic effects [20,21,40]. SC is a meiosis-specific protein structure that promotes synapsis (pairing of homologous chromosomes) and recombination events during the first meiotic prophase [72,73,74]. SC consists of two parallel lateral elements (LE), one central element (CE), and numerous transverse filaments (TF) (reviewed in [74]). The Synaptonemal Complex Proteins 2 and 3 (SYCP2 and SYCP3) are layered onto cohesin proteins producing the lateral elements of SC during the first meiotic prophase, whereas the transverse filament protein SCP1 [75] bridges the lateral elements at pachynema. Recent results have shown that the synaptonemal complex is required for both synapsis and the structural integrity of the chromosome axis [76]. Moreover, the repair of meiotic DSBs is impaired in the absence of SC in mice, highlighting the importance of this structure for the correct progression of meiosis in mammals [76]. Cytogenetic studies have used the synaptonemal complex as a biomarker for the genotoxic effects induced by IR, given that any chromosomal aberration can be detected when the SC is completely assembled during the pachytene stage [20,21,40,77]. Early studies performed by Allen and collaborators [78] detected three main types of SC abnormalities induced by chemical mutagens in male mice: (i) synaptonemal complex fragmentation, (ii) synaptic abnormalities and (iii) mispairing, or heterosynapsis. Later studies have identified other types of abnormalities, in most cases stage-dependent, of which the most common ones are chromosomal reorganizations that can be observed as circles, single or double, multivalents and loops [20,77,78]. Moreover, alterations in synapsis can also be also detectable, such as partial and/or complete asynapsis (reflected by the presence of univalents or bivalents with unpaired regions) or non-homologous pairing [19]. Very few studies have been focused on the radio-induced genotoxic effects in early stage of meiosis (oogonia and prophase I oocytes) and they are restricted to rat [19,20,39,40], mouse [21], Chinese hamster [33] and guinea pig [27]. Out of these, the studies performed in rat and mouse [19,20,21,39,40] have used SC as an indicator for genotoxic effects. 

## 4. Radiation-Induced Genotoxic Effects in Mammalian Female Germ-Cells

The literature is rather scarce in experiments *in vivo* in which female mammalian species were treated with IR and genetic alterations were analyzed in their germ cells or their progeny (Table 1). In general terms, irradiation studies performed on female mammalian germinal cells have demonstrated that radiation sensitivity is dependent on several factors, such as radiation dose (although the dose-effect relationship is poorly known), meiotic oocyte stage (taking into account the *granulosa* cells of the follicle), the parameter or measurable endpoint used and the species analyzed. Due to the intrinsic difficulties for the study of human meiosis, four main experimental model species have been used for radiation-sensitivity analysis (mouse, rat, guinea pig and Chinese hamster), resulting in a heterogeneous picture that makes it difficult to provide definitive conclusions by extrapolating results among species. As an example, when the parameters analyzed are chromosomal aberrations, DL and/or congenital malformations, studies have reported that oocyte radiation-sensitivity increased two-fold in the Chinese hamster, when compared to the mouse [34,35,61]. However, in the case of chromosome aberrations detected at MI/MII, the mature oocytes from guinea pig are more radio-sensitive than are mature mouse oocytes [23,79]. Despite this apparent disparity in results, depending on the species analyzed, it seems that a general trend can be outlined. In this section we will review the radiation-induced genotoxic effects studied in animal models by considering two different levels of analysis: (i) deleterious effects detected in maternal oocytes (F_0_) and (ii) the genotoxic effects detected in F_1_, through the analysis of trans-generational genome instability (chromosomal alterations and genetic mutations), aneuploidy, fertility and/or developmental malformations.

### 4.1. Analyzing the F_0_

Most of our knowledge regarding the radiation sensitivity of the female germ cells comes mainly from experiments performed in mice and, to a lesser extent, in rat, guinea pig and Chinese hamster. Radiation-induced genotoxic effects have been studied throughout all meiotic stages: oogonia, oocyte I (at leptonema, zygonema, pachynema or diplonema), immature oocytes (oocytes I at primordial or unilaminar follicles), maturing oocytes (oocytes I at pre- and antral follicles) and mature oocytes (oocytes I/oocytes II at pre- and ovulatory follicles) (Figure 1, Table 1). Moreover, there are also studies focused on post-conception stages, mainly at the zygote and pre-implantation stages (1-cell to blastocyst) and post-implantation stages (Table 1). All of these studies indicate that oocyte radiation-sensitivity depends on its developmental stage and its relation with folliculogenesis.

As already stated, few studies have focused on oogonia and prophase I oocytes [19,20,21,39,40]. Pujol and collaborators [19,20] analyzed the effect of different doses of X-rays (1 Gy, 2 Gy and 5 Gy) on the number of recoverable germ cells in female rat fetuses irradiated at three different days of gestation (d.g.): 14 d.g. (oogonia), 16 d.g. (leptonema) and 18 d.g. (zygonema). At 14 d.g., all germ cells are at the stage of mitotic proliferation, and as a result many oogonia are expected to be at the S phase of mitosis. At 16 d.g., many cells are completing their last mitotic division, and others have already entered the first meiotic prophase, whereas at 18 d.g. most cells have reached leptonema or lepto-zygonema. Their results indicate that irradiation significantly decreases the number of germ cells, and increases the incidence of synaptonemal complex fragmentation, suggesting that differences in sensitivity to irradiation depend on the meiotic stage at the time of irradiation. Differences in radiation doses were also observed; the 5 Gy dose is lethal for oogonia and zygotene oocytes, while for leptotene oocytes, this dose could have a sub lethal effect. Overall, Pujol and collaborators [19,20] show that SC fragmentation is stage-dependent; they found a significant increase in the incidence of heterosynapses in oogonia, a significant increase in the frequency of structural anomalies in leptotene oocytes and a significant increase in nuclear fragmentation in zygotene oocytes. Similar results were obtained by Johannisson and co-workers [21], when fetal female mice were exposed to ionizing irradiation of 2 Gy in a single dose at 14, 16, and 17 d.g., indicating that at early stages of meiosis, the genotoxic effects of IR are not species-dependent. 

Studies on immature and mature oocytes are more abundant in the literature. Overall, the observed general view is that mature oocytes are more radio-sensitive than are immature oocytes. These observations were already reported by early studies in mouse [16,37,46,54,55,65,80]. Later on, this trend was confirmed in other model species such as rat and guinea pig. Studies conducted in rat [39,40] showed that pre-pubertal dictiotene oocytes are less radio-sensitive than are post-pubertal dictiotene oocytes. In the same vein, and according to studies performed by Jacquet’s lab in guinea pig [23,30,79], immature oocyte are less radiation-sensitive than are maturing oocytes, and these are less radiation-sensitivity than are mature oocytes. On the other hand, it has been reported that the chromosomal sensitivity of immature dictiotene oocytes enclosed in primordial follicles is very low in the guinea pig [45]. In the late pre-antral stages the sensitivity of the oocyte gradually increases to reach a maximum at the antral stage of folliculogenesis. The same occurs in the Chinese hamster, where oocytes surrounding ovulation show the greatest sensitivity to radiation [62]. 

During oocyte maturation (between one day and six weeks after irradiation in the mouse), regardless of the fact that in all cases oocytes are arrested at the dictiotene prophase I stage, differences in radiation sensitivity have been reported. In mouse, ovulated oocytes (MII stage) analyzed one day to two weeks post-irradiation are less sensitive to the induction of chromosomal aberrations, genetic mutations and dominant lethal effects (DL) than are ovulated oocytes analyzed after three weeks and six weeks post-irradiation [47,57,80]. In particular, Tease [47] observed that at medium/high doses (100 cGy–600 cGy) radio-induced chromosomal aberrations increased two-fold in immature oocytes in relation to mature oocytes (Table 1). Ovulation is another key point. A few hours before ovulation, pre-ovulatory oocytes are more radio-sensitive, given that some studies have reported a significant increase in chromosomal aberrations [37,48,49,56] and chromosomal non-disjunction [81] in mouse irradiated at low doses (0.1 Gy–0.5 Gy). Analyzing DL, other authors have found that pre-ovulatory oocytes are radiosensitive in mouse [65,82] and rat [83]. Therefore, the sensitivity to the induction of genetic damage is dependent on the stage of folliculogenesis and, in this sense, the interval between irradiation and ovulation.

**Figure 2:**
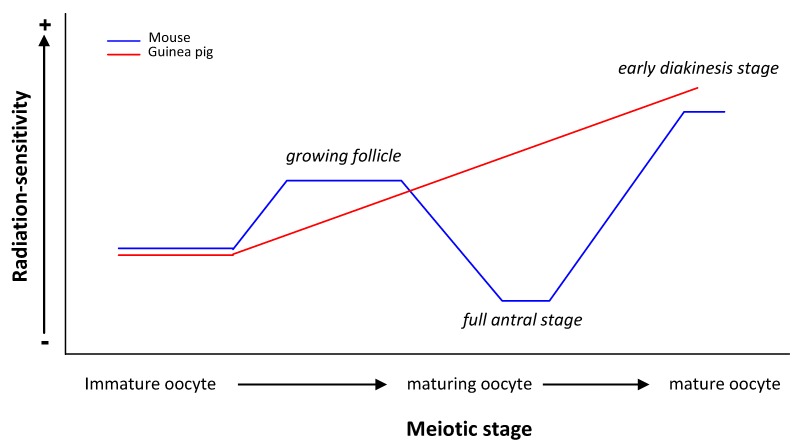
Comparison between genetic radiation-sensitivity observed in mouse and Guinea pig females.

At this point and taking into account published data on mammalian females (human included), we can consider that the genetic radiation-sensitivity observed in the human female is related to the size of the follicles in the way that small follicles are apparently less sensitive to cell-killing than are large follicles. In the case of mouse germ cells, radiation-sensitivity is related to the folliculogenesis stage (Figure 2) with its maximum sensitivity in early diakinesi (as seems to happen in Chinese hamster) and its minimum in the full antral stage. The few data published on the human female [11,12,16] differ from those obtained in guinea pig, where the maximum sensitivity would be in the full antral stage (Figure 2). Pre- and antral follicles have a larger (both in number and volume) granulose layer than do primordial follicles, probably acting as a protective screen against the genotoxic effects of radiation. Therefore, why, in some species, are mature oocytes more radio-sensitive than are immature oocytes? One has to take into account that a follicle works as a functional syncytium, so the response may lie in how the DNA damage-response changes during oocyte development and how they are challenged by ionizing irradiation. Moreover, we have to bear in mind, however, that all parameters used in the literature to measure radiation-induced genotoxic effects are highly diverse (from chromosomal alterations to cell-killing and congenital malformations) and, therefore, the repair mechanisms involved might be different so, therefore, we have to be extremely cautious when extrapolating the data. Notwithstanding these differences, a wide variety of direct and indirect evidence indicates that oocytes maintain their capacity to repair DNA insults during their maturation process [84,85,86,87,88,89,90]. In a recent study, Wang and collaborators [91] described the proteome of mouse oocytes at different developmental stages, the dictiotene or germinal vesicle (GV) stage, the metaphase II (MII) stage and zygote stage (fertilized oocyte). Surprisingly, the authors found that more DNA repair proteins are expressed in MII oocytes than in GV oocytes or the zygote; they included in the analysis the proteins involved in the SSB repair and nucleotide-excision repair pathways. Moreover, they found that the DNA recombination pathway and DNA replication protein families are also more abundant in MII oocytes. In particular, 35 out of 53 proteins involved in the DNA repair process identified in both MII oocytes and zygotes are up-regulated in the MII oocytes. These include Rad51, BRCA2, XRCC1, PARP1 and TRIP13. These results are consistent with previous findings on gene expression profiles of oocytes and early embryos [92], which suggest that the over-representation of genes involved in DNA repair confer protection to maintain genome stability of the female germ-line.

Based on the evidence, a possible interpretation of differences in radiation-sensitivity during oocyte maturation is that mature oocytes repair genetic insults by means of “prone to error” mechanisms, stabilizing alterations through chromosomal rearrangements. That would explain why chromosomal aberrations are frequently observed at MII [37,49,50,93]. On the contrary, immature oocytes would not efficiently repair the genetic insults, resulting in cell death and not contributing to the offspring. Species differences to the genotoxic effects of IR can also be attributed to differences in the implementation of DDR and, therefore, more studies are needed to overcome any disparity in reports.

### 4.2. Trans-Generational Studies

When an increased rate of mutation or augmentation of developmental malformations, to name two different measurable endpoints, is observed in the non-exposed offspring of irradiated parents, we are facing trans-generational genomic instability [37,49,50,93]. According to Barber and Dubrova [94], the first evidence of a trans-generational effect for ionizing radiation was demonstrated by Luning *et al.* [95], who observed elevated rates of dominant lethal mutations in the non-exposed first-generation (F_1_) offspring of males injected with 239^Pu^ citrate solution. Data on potential trans-generational genomic instability following irradiation are largely limited to males [94,96,97,98,99], and only until very recently did studies in females begin to flourish [39,40,41,42,43,51,60]. Early studies evaluated the genotoxic effects in prophase I stages (pachynema, diplonema and dictionema oocytes) in the Chinese hamster [33,34,35]. After exposing animals to a single dose of 1 Gy, the authors did not observe a significant increase of chromosomal aberrations in one-cell embryos and/or developmental alterations among the progeny of the irradiated females. Therefore, and although these studies detected that radio-resistance was stage-dependent, they did not observe any trans-generational effects with these measurable endpoints. Subsequent studies in mice by Pils and collaborators [22] studied the genotoxic effects when irradiating females at the zygote stage. They not only obtained positive results when looking for fertility alterations of these irradiated females, but they also found an increment of congenital malformations in the F_1_ progeny. Nevertheless, years later, Jaquet and co-workers [24] reported negative findings regarding developmental defects and genomic instability (analyzing chromosomal aberrations) in the F_1_ and after exposure of low doses of irradiation at the mouse female zygote stage (0.2 and 0.4 Gy instead of 1 Gy used by Pils *et al.* [22]). Here, the authors suggested differences in the mouse strain and sex-radiation sensitivities. Interestingly, studies leaded by Dubrova’s group [51,60], based on the analysis of expanded simple-tandem repeats (ESTR), indicate that whereas paternal irradiation destabilizes the genomes of non-exposed offspring, maternal irradiation does not affect stability of their offspring. Taking together, these data show that the trans-generational effects in the offspring of irradiated male mice may be explained by a genome-wide destabilization, which manifests in many, possibly all, tissues. Why this behavior regarding ESTR is not observed in females needs further validation.

Others have studied trans-generational genomic instability through the analysis of chromosomal alterations detected in fibroblasts derived from fetuses, which resulted from fertilization of irradiated oocytes [25,26,39,40,41,42,43]. In their initial studies using mouse as a model species, Pampfer and Streffer [25] assumed that the observed structural chromosomal aberrations were not induced directly by radiation exposure and the radiation exposure of the zygote has led to instability of the genome so that “spontaneous” aberrations occurred more frequently. In doing so, these authors had already detected an increase in trans-generational chromosomal aberrations in mouse zygotes and, although these former results were not totally conclusive, “the intuited” trans-generational chromosomal instability in mouse was corroborated by Streffer, [26] some years later. To our knowledge, there is only one published work in mouse with trans-generational positive results [49]. In this case the statically significant F_1_ chromosomal aberrations only correspond to pre-implantation stages as if the chromosomal aberrations were life-incompatible with later stages.

### 4.3. The Rat as a Model Species

Mirroring the commented effects in mouse, studies in rat performed by our group came to similar conclusions. The rat has been used as a model species for evaluating the radiation-induced genotoxic effects in mammalian female germ cells, either in the irradiated females or in the offspring [19,20,39,40,41,42,43]. In general terms, studies of trans-generational radiation-induced genotoxic effects on the gonadal function of rat females, using mainly SCs or chromosomal alterations as measurable endpoint markers, have concluded that, in the case of F_0_ fetal oocytes, cell-killing is dose-dependent and that SC fragmentation is prophase-stage-dependent [19,20]. Moreover, no trans-generational cytogenetic effects were observed in the progeny of irradiated females, indicating that those oocytes that remain after irradiation do not present genome instability or any constitutional aberration in the SCs. The same occurs when studying the F_1_ fetal oocytes from pre-pubertal and adult irradiated mothers by SCs where the remaining oocytes do not present either genome instability or any constitutional aberration, although there is an induction of apoptosis in the fetal rat germ cells [39,40]. More recently, we studied fibroblasts derived from fetuses of irradiated mothers [41,42,43], and in this case, a radiation-induced chromosomal instability was observed (an increase of chromosomal instability but not constitutional chromosomal alterations) in the offspring of irradiated females. In our view, this apparent contradiction to previous data [39,40] suggests that the use of chromosomal parameters and not just the analysis of SCs is more efficient in detecting germ cell genomic instability.

## 5. Human Females: An Approachable Model? Future and Prospects

Most of the current knowledge of the genotoxic effect of IR in the human female germ cell line came from epidemiological studies among atomic disaster survivors (*i.e.*, Chernobyl), radiation workers or cancer patients that have been submitted to radiotherapy (reviewed in [53]). But we have seen that data on mammalian females are controversial so far due to the disparity in type of irradiation, doses employed, parameters analyzed (chromosomal reorganizations, congenital effects, frequency of microsatellite mutations, among others), age and exposure to environmental factors. Moreover, and due to the intrinsic characteristics of human female gametogenesis (*i.e.*, progression during fetal development), its study has been limited to sampling availability. Only until very recently have new efforts been directed to the development of an *in vitro* system that allows meiotic progression in a sytematically and reproducible way; this technique in the future will allow us to molecularly study the evolution of the human female meiotic prophase in a systematic way, thus avoiding the side-effects of *in vivo* studies. Our group and others have worked on establishing the conditions that will allow female meiotic progression *in vitro* [100,101,102,103,104,105,106,107,108]. Some of them have indeed used organotypic culture models to investigate the radio-sensitivity of early female proliferating germ cells [107]. However, the method developed recently by Brieño-Enríquez and co-workers signifies a breakthrough in the study of meiosis, since it is the first culture system that allows mammalian meiocytes to continue meiosis *in vitro* ever reproduced. This method represents a new way to test the toxicity of chemical [106,109] as well as physical [110] agents to be used in humans, opening new avenues for the study of oogenesis development and regulation in female reproduction. 

## 6. Conclusions

In summary, irradiation studies performed on germinal cells from mammalian females have demonstrated that radiation sensitivity is a complex affair. Oocyte radiation sensitivity is always dose-dependent, although there is no doses-effect relationship described. Usually, radiation sensitivity is dependent on the meiotic/oocyte-irradiated stage and, in some cases, species-dependent. Overall, we can consider that the genotoxic effects are dependent on the parameters analyzed and could be considered to be stage-dependent.
